# Superior gluten structure and more small starch granules synergistically confer dough quality for high amylose wheat varieties

**DOI:** 10.3389/fnut.2023.1195505

**Published:** 2023-05-17

**Authors:** Liqun Li, Zhenzhen Liu, Xu Li, Xiangnan Chu, Weibing Yang, Bingxin Wang, Yanzhou Xie, Xuejun Li

**Affiliations:** State Key Laboratory of Crop Stress Biology in Arid Areas and College of Agronomy, Northwest A&F University, Yangling, Shaanxi, China

**Keywords:** gluten structure, starch granule development, high amylose wheat, starch properties, dough quality

## Abstract

High amylose wheat (HAW) has potential health benefits but its dough structure is usually inferior. Wheat dough is a complex mixture and its structure is influenced by the physicochemical properties of gluten and starch. In this study, we investigated the starch granule development, gluten structure, starch properties, pasting, and thermal properties of flour, as well as the rheological properties of dough in wheat variety Xinong 836 with high amylose content (33.57%) and its parents. The results showed that Xinong 836 wheat starch contained more small starch granules, which was consistent with the microstructural results of starch granules in grain filling stage. Moreover, Xinong 836 wheat starch showed highest swelling power and water solubility. Importantly, the flour of Xinong 836 wheat had the highest protein content and wet gluten content and Xinong 836 wheat gluten showed highest β-sheets content and disulfide bond content than its parents Zhengmai 7698 and Xinong 979, which conferring to more compact microscopic networks of dough, thereby contributing to the higher peak viscosity (PV), final viscosity (FV), and setback viscosity (SB) in the flour of Xinong 836. Our finding elucidated that the stability of gluten and properties of starch synergistically affected the pasting and thermal properties of the flour paste, and the presence of more small starch granules contributed to dough with a rather dense structure in HAW Xinong 836. Thus, superior gluten structure and more small starch granules have synergistic effects on enhancing the gluten–starch interaction, thereby contributing to better dough quality.

## Highlights

- Xinong 836 had high amylose content and contained more small starch granules in later endosperm development stages.
- Presence of more small starch granules resulted in dough with a rather dense structure.
- Starch relative crystallinity negatively correlated with viscosity parameters and positively correlated with enthalpy.
- Stable gluten network beneficial for enhancing shear force resistance of flour paste.

## 1. Introduction

Wheat (*Triticum aestivum* L.) is one of the most important cereal crops in the world and is widely used in the production of various flour-based foods. Storage proteins and starch are the main components of wheat flour, and they are accumulated in the endosperm during grain development and stored in protein bodies (PBs) and starch granules (SGs), respectively. Upon addition of water and next stirring, wheat flour can form the dough with a unique three-dimensional structure and viscoelasticity woven by gluten and starch. Gluten proteins constitute 80–85% of wheat flour proteins and are considered to have crucial effects on the properties of dough (1). Native gluten can be divided into soluble gliadin and insoluble glutenin. Gliadins and glutenins are mainly connected by intramolecular and intermolecular disulfide bonds to form a gluten network structure. Meanwhile, glutenins are further divided into high-molecular-weight glutenin subunits (HMW-GSs) and low-molecular-weight glutenin subunits (LMW-GSs) based on the molecular weight (2). The secondary structure of gluten is critical for determining the quality of dough, in particular, the β-sheets structure content is positively correlated with the viscoelasticity of dough (3). Therefore, the β-sheets and disulfide bonds in the gluten structure synergistically affect the formation of a dense and stable gluten network structure.

Another important component of wheat is starch, which is composed of slightly branched amylose and highly branched amylopectin. Starch usually comprises approximately 25% amylose and 75% amylopectin (4), while the amylose contents of some wheat varieties are higher than typical wild-type lines, and they are referred to as high amylose wheat (HAW). Starch granule (SG), the primary form of starch in wheat endosperm, is mainly in the range of less than 1 μm to more than 100 μm and can be divided into A-type (>10 μm) and B-type (<10 μm) according to their size (5, 6). It is now generally accepted that different morphologies of starch granules among the wheat varieties were due to different amylose content, such as the higher amylose content wheat having more B-type starch granules while higher amylopectin content wheat owing more A-type starch granules (6, 7). The starch hydrolysis rate was slowed and the extent was reduced when amylose content increased, and thus the HAW varieties contributing significantly to the maintenance of human health (8). Recent studies show that starch plays a more and more important role in the properties of dough. Starch, together with other minor ingredients, is embedded in the gluten network. The physicochemical properties of starch including the particle size, relative crystallinity, and pasting properties affect flour quality, dough functionality, and the sensory properties of food products (9).

Several studies have attempted to formulate food products by mixing HAW starch with other commercial wheat flour. A recent study indicated that the addition of HAW starch would lead to lower bread volumes (10). Coincidently, the other report suggested that increasing proportions of HAW starch reduced the viscosity, compression stress and elasticity of dough when blended with commercial hard wheat flour (11). Another study showed that increasing the amylose content resulted in greater hardness, fewer pores, and a lower specific volume of bread (12). Besides, initial crumb firmness increased with higher levels of amylose content was also found in the previous study reported by Nivelle et al. (13). It thus appears that HAW flour generally produces inferior dough and leads to a decreased quality in final baked products. Further studies showed that this decreased quality might be related to the extent of the gluten protein dilution and a hindrance effect on the gluten network development by the non-gelatinized HAW starch granules (10), whereas the deep could be due to the factor that HAW starch granules possess a more irregular shape and rougher surface compared to normal wheat starch granules, and the aggregation of starch granules was more pronounced in the dough, leading to the poor dispersion of HAW starch granules in the protein phase (14). Up to now, the effects of gluten on the dough quality in normal wheat, including those of different glutenin subunits, various enzymatic modifications, and physicochemical treatments have been investigated (15, 16). However, few studies have been focused on the interaction between gluten and starch of native HAW and their effects on the properties of dough.

Wheat variety Xinong 836 is characterized by a higher amylose content compared with normal wheat varieties. Xinong 836 and its parents are widely planted in China, and thus they were investigated as experimental materials in the present study. In particular, we investigated starch granules development, the gluten structure, starch properties, and dough mixing properties in these wheat varieties. Furthermore, we analyzed the effects of gluten, starch, and their interaction on the behavior of dough. Our results provide useful insights into the physicochemical properties of gluten and high amylose starch, and the effects of their interaction.

## 2. Materials and methods

### 2.1. Plant materials

Three wheat varieties comprising Xinong 979, Zhengmai 7698, and Xinong 836 were used in this study. Xinong 836 is the progeny obtained by crossing Xinong 979 and Zhengmai 7698. The F1 progenies were created by initially crossing Xinong 979 and Zhengmai 7698. According to the pedigree method, the selection of desirable single plants was conducted from F2 through several generations, and the selection including the determination of various HMW-GSs compositions and amylose content. After six generations of continuous selfing and selection, the homozygous wheat lines Xinong 836 was obtained and approved by Shaanxi Provincial Wheat Variety Committee in 2019.

The three wheat varieties were planted individually in rows at Yangling (108°40′E, 34°16′N), Shaanxi Province, China, during two consecutive wheat growing seasons (2018–2019 and 2019–2020). The plot size was 20 × 2 m × 0.23 m and the row spacing was 0.23 m. Three biological replicates were performed for each wheat variety. The spikes obtained from three varieties were marked at the flowering stage. About 50 labeled spikes were randomly collected from each wheat variety at 7, 16, and 28 days after anthesis (DAA). The labeled spikes were used for cytological observations. After harvesting, the wheat grains were milled using a multifunctional pulverizer (Y400, Laobenxing, Zhejiang, China), and passed through a 100-mesh screen.

### 2.2. Microstructural endosperm observations in the grain filling stage

Wheat kernels were collected at 7, 16, and 28 DAA to observe the microstructure of the caryopses. Slices of the wheat endosperm were prepared and immersed immediately in 4% glutaraldehyde fixative at 4°C for 8 h. The samples were subsequently washed three times with 0.1 M sodium phosphate buffer (pH 6.8). Finally, the slices were finally embedded in white resin, then carefully cut into 1-μm slices by using a histotome (RM2265, Leica Microsystem Ltd., Wetzlar, Germany) and stained with toluidine blue (0.03 M) for 10 s. The samples were observed under a Leica microscope (DMLS, Leica Microsystem Ltd., Wetzlar, Germany).

### 2.3. Assessment of grain quality traits

A near-infrared reflectance spectrometer (Diode Array 7250, Perten, Stockholm, Sweden) was used to obtain the quality-related parameters for the mature wheat grains as described by Gao et al. (9). As the relevant spectral regions show reasonably clear differences with changing sample composition, NIR can predict some parameters with a high degree of accuracy, including the moisture, starch, protein, and wet gluten contents, and the Zeleny sedimentation value. The mean values were calculated based on three individual tests.

### 2.4. Extraction and separation of gluten and starch

Dough samples were prepared by mixing flour with water [flour (g): water (mL) = 5:3], followed by recovery for 15 min. Gluten and starch were separated by hand washing. The gluten was lyophilized in a freeze dryer (ScanVac CoolSafe, LaboGene, Scandinavia peninsula, Denmark), ground into a fine powder, passed through a 100-mesh sieve, and finally stored at 4°C. The starch slurry was successively washed with 2 M sodium chloride, 2% sodium dodecyl sulfate (SDS), and distilled water and centrifuged. And this process was repeated three times. Finally, the pure starch was oven-dried, passed through a 100-mesh sieve and stored at 4°C.

### 2.5. Determination of glutenin composition and structural properties of gluten

Glutenin samples were extracted and HMW-GS were detected by SDS-polyacrylamide gel electrophoresis. The secondary structure of gluten can be determined based on the amide I band (1,600–1,700 cm^−1^) in the Fourier transform infrared (FTIR) spectra as reported by Song et al. (17). The gluten samples were pressed into thin sheets. An FTIR spectrometer (Vertex 70, Bruker, Germany) was used to record the FITR map at 25°C with a spectral resolution of 4 cm^−1^. The main structures comprised intermolecular β-sheets at 1,612–1,620 cm^−1^, β-sheets at 1,625–1,642 cm^−1^, α-helices at 1,650–1,660 cm^−1^, β-turns at 1,670–1,680 cm^−1^, and antiparallel β-sheets at 1,680–1,695 cm^−1^. The area under each sub-peak was calculated and the corresponding secondary structure content was obtained as follows: Secondary structure content = Secondary structure sub-peak area/total area of amide I region. The sulfhydryl groups and disulfide bonds were determined according to the method reported by Zhu et al. (18). The following is a detailed description. First, 30 mg of the freeze-dried sample was dispersed in 8 mL of Tris-Gly buffer, the sample was agitated and extracted at room temperature for 1 h, and then the supernatant was collected by centrifugation at 12,000 × g for 10 min. In order to measure the free SH contents (SHfree), 0.1 mL of 10 mM 5,5′-dithiobis-(2-nitrobenzoic acid)5 (DTNB) were added to 4 mL supernatant. After reacting for 20 min at room temperature, determine the absorbance at 412 nm. In order to measure the total SH contents (SHtotal), 0.1 mL of β-mercaptoethanol and 4 mL of Tris-Gly buffer were added to 1 mL supernatant, and the mixture was incubated for 1 h at 25°C. Next, 4 mL of trichloroacetic acid (12%, w/v) was added to the mixture, before incubating for another 1 h at 25°C. The mixture was centrifuged at 3,000 × g for 20 min and the supernatant was removed. This procedure was repeated three times. The final precipitate was collected and dissolved in 10 mL of 0.2 M Tris-Gly buffer, and 0.1 mL of 10 mM DTNB was added. Absorbance was determined following the above method. The free SH and total SH contents of each sample were calculated as follows, the disulfide bond content was calculated from SHfree and SHtotal.


SHcontent(μmol/g)=73.53×A412×D/C.

−S−S−content(μmol/g)=(SHtotal−SHfree)/2.

Where A412 is the absorbance at 412 nm, D is the dilution factor, i.e., 5.02 for free SH and 10 for total SH, C is the sample concentration (mg/mL), and 73.53 is derived from 10^6^/(1.36 × 10^4^), where 1.36 × 10^4^ is the molar absorptivity. The experiments were repeated independently in triplicate.

### 2.6. Starch properties

#### 2.6.1. Determination of amylose contents and morphology of SGs

The amylose contents were determined using the dual-wavelength iodine binding method reported by Liu et al. (19) with a slight modification. In brief, the 100 mg sample was suspended in 10 mL 0.5 mol/L KOH. The suspension was heated in boiling water for 10 min with shaking and cooled to room temperature. And the volume was made up to 50 mL using distilled water. Next, 2.5 mL of aliquot was transferred to 50 mL beaker, adjusted pH value to 3.5 with 0.1 mol/L HCl, add 0.5 mL iodine reagent (20 g potassium iodide and 2 g resublimed iodine were dissolved in 100 mL distilled water), then made up to 50 mL with distilled water. The mixture was kept at room temperature for 30 min. Then, the absorbance was measured at the detection wavelength and reference wavelength and further converted into amylose and amylopectin content according to the standard curve. The standard curve is generated by using the reference samples of amylose (A8160, Solarbio, Beijing, China) and amylopectin (A8150, Solarbio, Beijing, China). Measurements were repeated three times for each sample.

#### 2.6.2. Morphology and size distribution of SGs

The morphology of SGs was observed by scanning electron microscopy (SEM, JSM-6360LV, JEOL, Japan). Starch samples were placed on a metal stub with double-sided adhesive tape and sprayed with gold particles for 90s in a vacuum evaporator. Scanning electron micrographs were captured using SEM at a magnification of 1,000×. The number and volume distribution were determined for SGs using a Microtrac S3500 laser diffraction instrument (Microtrac S3500 SI, Microtrac Inc., Montgomeryville, United States) and laser diffraction particle size analyzer (Mastersizer 2000E, Malvern, United Kingdom), respectively.

#### 2.6.3. Swelling power and solubility

Each starch sample was weighed and placed in a 2 mL centrifuge tube, before mixing with water to form a starch solution (2%, w/v). The starch solution was heated in water at 95°C for 30 min and shaken gently. The tubes were cooled to room temperature, centrifuged at 8000 × *g* for 10 min, and the sediment was weighed. The sediments in the tubes were then dried to a constant weight and weighed again. The swelling power and solubility were calculated as described by Zhu et al. (20). The experiments were repeated in triplicate.

#### 2.6.4. X-ray diffraction (XRD) analysis of starch

XRD patterns were acquired for starch samples using an X-ray diffractometer (D8-ADVANCE, Bruker, Germany). The starch samples were scanned at 30 mA and 40 kV, with diffraction angles (2θ) ranging from 4° to 40° at a scan rate of 2° min^−1^. The relative crystallinity was calculated by using MDI Jade 6.0 software (Materials Data, Inc., Livermore, CA).

### 2.7. Pasting properties and thermal properties of flour

The pasting properties of flour were determined using a rapid viscosity analyzer (RVA 4500, Perten, Stockholm, Sweden). A sample weighing 3 mg and 25 g of ultrapure water were added to the canister. The pasting parameters including the peak viscosity (PV), breakdown viscosity (BD), final viscosity (FV), setback viscosity (SB), and pasting temperature (PT) were obtained using the pasting program described by Shang et al. (21).

The thermal properties of the samples were determined using a simultaneous thermogravimetric analyzer (STA7200RV, Hitachi, Tokyo, Japan) according to the method described by Song et al. (17). The onset, peak, and conclusion temperatures (T_o_, T_p_, and T_c_), and enthalpy of thermal transition (ΔH) were obtained using TA7000 software (version 10.41, Hitachi, Tokyo, Japan). All of these measurements were repeated three times.

### 2.8. Mixing properties of dough

The dough development time and stability time were analyzed using a Mixolab2 system (Chopin, Tripette and Renaud, France), and calculated using the Mixolab “Chopin +” protocol. Each flour sample was tested independently in triplicate.

### 2.9. Confocal laser scanning microscopy (CLSM) analysis of dough

The dough samples were post-stained using a dye mixture comprising 0.010% (w/w) rhodamine B solution in water. The microstructure of each dough sample was observed by CLSM as described by Gao et al. (9). For each dough sample, 10 images of gluten were captured. With the assistant of AngioTool64 version 0.6a (National Cancer Institute, National Institute of Health, Maryland, United States), the gluten microstructure was quantified.

### 2.10. Statistical analysis

Differences among data were detected using one-way analysis of variance (SPSS version 21.0, SPSS Inc., Chicago, IL, United States), and significant differences among parameters were compared at *p* < 0.05 with Duncan’s multiple range test. Pearson’s correlation coefficients were calculated between the parameters.

## 3. Results and discussion

### 3.1. Basic components

The basic components of the three wheat varieties are shown in Table 1. Xinong 836 wheat starch had the highest amylose content (33.57%), protein content (14.40%) and wet gluten content (29.87%) compared with Xinong 979 and Zhengmai 7,698. According to Tatsuya et al. (22), wheat with amylose content greater than 30% was classified as HAW. And some studies obtain HAW whose amylose content is higher than 50% by using the genetic strategy (4). However, most previous studies are solely concerned with amylose content and the functional properties of HAW starch. And the gluten components in HAW have rarely been investigated.

**Table 1:** Quality related parameters for Xinong 979, Zhengmai 7698, and Xinong 836.

Variety	Moisture (%)	Protein (%)	Wet gluten (%)	Starch content (%)	Amylose content (%)	Dough development time (min)	Dough stability time (min)
Xinong 979	8.44 ± 0.03c	13.74 ± 0.04b	28.63 ± 0.01b	81.89 ± 0.02a	24.67 ± 0.60b	6.81 ± 0.21a	9.05 ± 0.19b
Zhengmai 7698	8.94 ± 0.06a	12.46 ± 0.05c	26.97 ± 0.01c	80.82 ± 0.03b	24.32 ± 0.18b	4.16 ± 0.18b	5.23 ± 0.14c
Xinong 836	8.74 ± 0.08b	14.40 ± 0.18a	29.87 ± 0.43a	80.17 ± 0.29c	33.57 ± 0.44a	7.16 ± 0.23a	9.55 ± 0.07a

^1^Values in the same column followed by different letters are significantly different (*p* < 0.05).

In general, it is considered that higher protein content improves the end-product quality. According to previous studies, gluten proteins comprise 80–85% of wheat flour proteins, and greater amounts of gluten may enhance the formation of the gluten network (1). Thus, the wheat variety Xinong 836 has a higher amylose content but also the potential to form a strong gluten network. And we carried out the follow-up study.

### 3.2. Cell morphology observations in the grain filling stage

**Figure 1:**
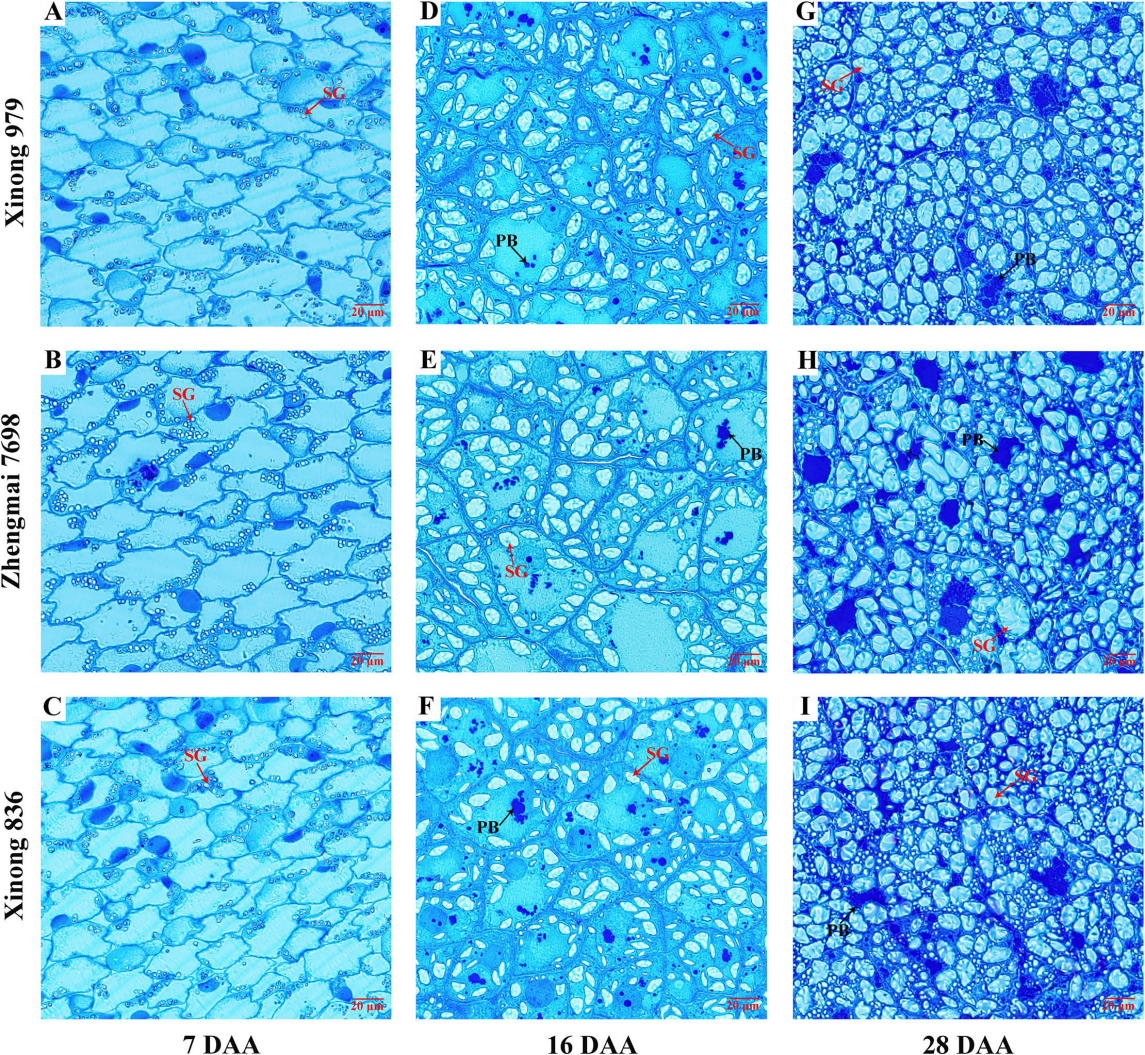
Microstructural of wheat endosperm in the three wheat varieties. Microstructural images of the wheat endosperm at 7, 16, and 28 days after anthesis (DAA) in Xinong 979 **(A,D,G)**, Zhengmai 7,698 **(B,E,H)**, and Xinong 836 **(C,F,I)**. Scale bar, 20 μm. PB, protein body; SG, starch granule.

The accumulated PBs and SGs in endosperm cells in the three wheat varieties were observed at 7, 16, and 28 DAA using optical microscopy. During the early filling stage (7 DAA), PBs did not appear to be accumulated in the three varieties, but SGs formed in the endosperm and they were distributed along the cell wall (Figures 1A–C). PBs were clearly observed at 16 DAA and most appeared to be aggregated. The number and size of SGs also increased at this stage (Figures 1D–F). Figure 1F shows that the increase in PBs was more obvious in Xinong836. The size and number of PBs and SGs increased further at 28 DAA. Most of the PBs aggregated together to form an amorphous protein matrix and they remained in the gaps between SGs, while more small SGs appeared (Figures 1G–I). Remarkably, the PBs tended to be dispersed randomly among the SGs, and more small SGs were present in the endosperm cells in Xinong 836 (Figure 1I). The results indicated that the accumulation of protein and starch during grain filling stage is different among the three wheat varieties. And this may predicts that differences in the accumulation of protein and starch during grain filling stage possibly affected the mature grain, such as the protein structure and starch properties.

### 3.3. Glutenin composition and gluten structure

The HMW-GS compositions were determined in the three test varieties and compared with known subunit combinations in control wheat lines (Supplementary Figure S1). Xinong 836 and Zhengmai 7698 had the same subunit combination (Ax1, Bx7, By9, Dx5, and Dy10), but they differed from that in Xinong 979 at the *Glu-B1* and *Glu-D1* loci. Relating the subunit composition to certain quality traits is a frequently used methodology in wheat. The Payne score provided a single-number parameter to estimate the dough strength of a sample as a function of its HMW-GS allelic composition (23). The Payne scores for the three varieties were ranked as follows: Xinong 836 = Zhengmai 7698 > Xinong 979. However, the Payne score based on the HMW-GS composition alone cannot accurately reflect the strength of the gluten network and predict the behavior of dough. Thus, we studied the gluten structure in the three wheat varieties. The gluten secondary structures in the three wheat varieties are shown in Table 2. Xinong 979 and Xinong 836 had higher intermolecular β-sheets contents. The β-sheets contents in the three wheat varieties ranged from 33.79% for Zhengmai 7698 to 34.60% for Xinong 836, whereas the α-helix contents followed the opposite trend. In addition, Xinong 836 had the highest disulfide bond content (36.56 μmol g^−1^). It is widely accepted that β-sheets and intermolecular β-sheets contribute to a more stable gluten network conformation (17). However, the α-helix content is negatively correlated with dough stability (15). Moreover, disulfide bonds are positively correlated with the accumulation of protein polymers (24). Thus, the accumulation of gluten during grain development had important effects on the structural properties of wheat gluten. In general, disulfide bonds and β-sheets have a synergistic effect on the structure of gluten, and thus Xinong 836 produced an excellent gluten network.

**Table 2:** Gluten structure and starch properties for Xinong 979, Zhengmai 7698, and Xinong 836.

Variety	Gluten structure							Starch properties				
	Intermolecular β-sheets (%)	Antiparallel β-sheets (%)	α-helices (%)	β-sheets (%)	β-turns (%)	Sulfhydryl groups (μmol/g)	Disulfide bonds (μmol/g)	AC (%)	BC (%)	Relative crystallinity (%)	Swelling Power (g/g)	Water solubility (%)
Xinong 979	8.15 ± 0.06a	14.13 ± 0.04b	23.83 ± 0.02b	34.13 ± 0.13ab	19.76 ± 0.06b	15.52 ± 0.06b	33.85 ± 0.14a	35.00 ± 1.59a	65.00 ± 1.60c	24.96 ± 0.11a	20.58 ± 0.08c	23.73 ± 1.68b
Zhengmai 7698	3.70 ± 0.82b	15.32 ± 0.38a	26.25 ± 0.25a	33.79 ± 0.10b	20.94 ± 0.30a	21.03 ± 2.78a	22.67 ± 3.75b	30.05 ± 3.53b	69.95 ± 3.53b	22.81 ± 0.05b	24.40 ± 0.05b	22.38 ± 0.53b
Xinong 836	8.07 ± 0.20a	13.89 ± 0.08b	23.74 ± 0.29b	34.60 ± 0.36a	19.70 ± 0.18b	14.15 ± 0.49b	36.56 ± 2.37a	23.06 ± 1.01c	76.94 ± 1.01a	21.38 ± 0.13c	26.23 ± 0.05a	27.15 ± 0.34a

^1^Values are expressed as mean ± standard deviation (*n* = 3). Results followed by a different letter in the same column are significantly different (*p* < 0.05).

^2^AC, A-type starch granule content; BC, B-type starch granule content.

### 3.4. Starch properties

**Figure 2:**
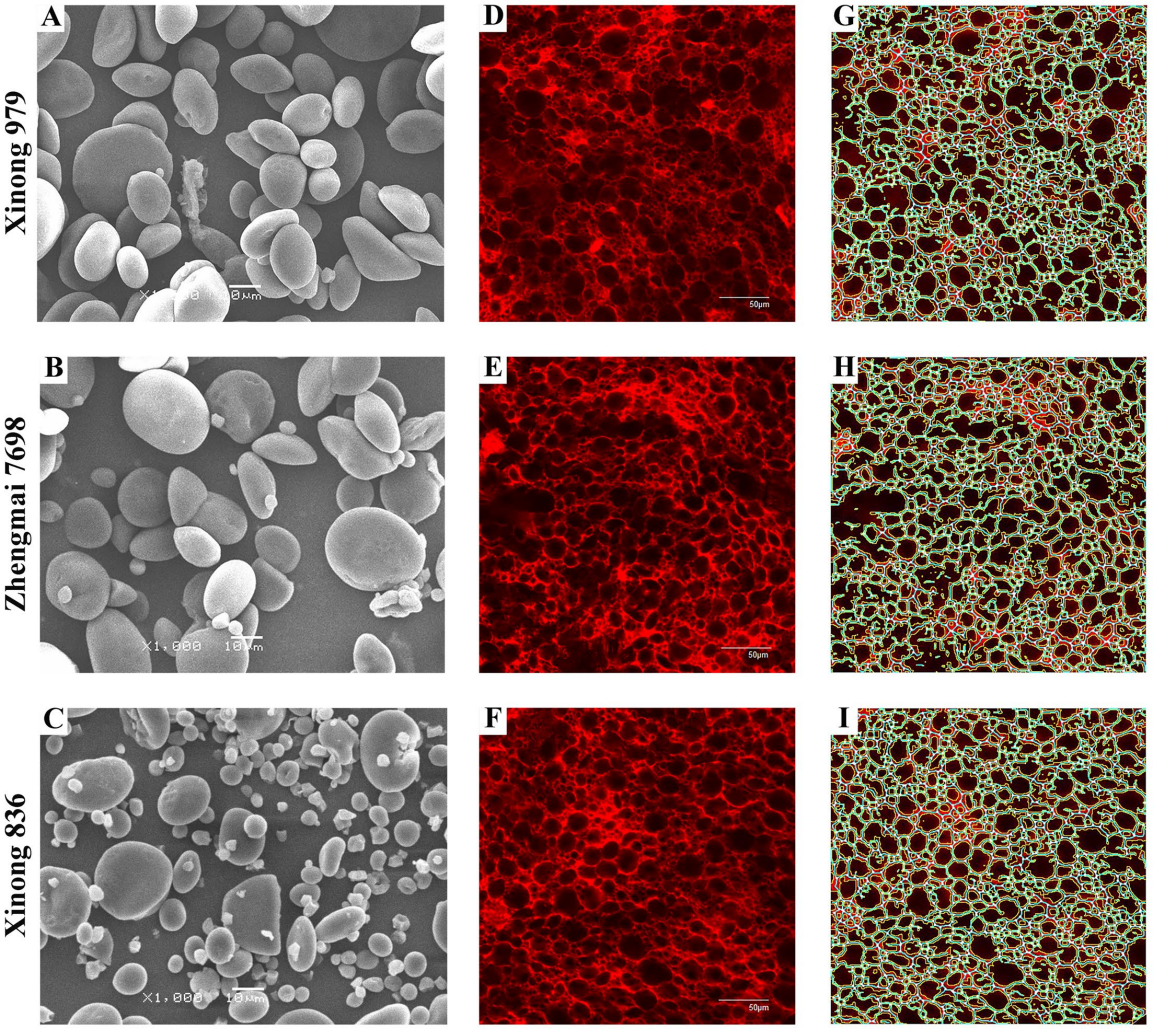
Starch granules and the protein network structure in the three wheat varieties. Images of isolated starch granules and the protein network structure in dough samples produced from Xinong 979 **(A,D,G)**, Zhengmai 7698 **(B,E,H)**, and Xinong 836 **(C,F,I)**. **(A–C)** Morphology of starch granules observed by scanning electron microscope at a magnification of 1,000×. **(D–F)** Original stained images of dough obtained by laser scanning microscopy, where the scale bar represents 50 μm. **(G–I)** Gluten network structure processed with AngioTool, where white represents the junctions, blue represents the protein skeleton, yellow represents the protein outline/area, and the black area is filled with starch granules.

We used SEM to observe the morphology of the SGs in the three wheat varieties, including large disk-shaped or lenticular A-type SGs, and polyhedral, irregular, and oval B-type SGs (Figures 2A–C). Previous reports indicated that amylose content was positive relative with large SGs (25). However, accumulating evidences showed that smaller SGs contributed to higher amylose content (14, 26, 27). Similarly, in the present study, the HAW Xinong 836 contained more irregular SGs and small SGs (Figures 2A–C).

The number distributions of different SGs types in the samples varied greatly (Table 2 and Supplementary Figure S2A). Xinong 979 had the highest A-type SGs content (35.00%), whereas Xinong 836 had the lowest (23.06%). The B-type SGs content varied from 65.00% in Xinong 979 to 76.94% in Xinong 836. Among the three wheat varieties, Xinong 836 contained more small SGs, which was consistent with the SEM observations (Figure 2C) and the images of SGs accumulated in the endosperm (Figure 1I). This indicates that the differences in the accumulation of starch during grain filling stage ultimately leads to different starch granule size in mature grains. From the perspective of endosperm development, wheat with increased amylose content is accompanied by smaller starch granules.

Supplementary Figure S2C shows that the XRD patterns contained four prominent diffraction peaks at 15°, 17°, 18°, and 23°. The results demonstrated that the typical A-type crystal structure was found in the three wheat varieties. In particular, Xinong 836 had the lowest relative crystallinity (21.38%) and it differed significantly from those in Xinong 979 and Zhengmai 7698 (Table 2). According to Kim and Huber (7), higher content of B-type SGs is relative to shorter amylopectin branch-chains but less content of intermediate and long branch-chains, which leads to the sharper intensity and lower relative crystallinity of starch. Meanwhile, Li et al. (28) found that the crystallinity decreased as the amylose content increased. Regina et al. (29) found that as the amylose content increases the semi-crystalline structure of starch gets disrupted, as evidenced by loss of crystallinity and birefringence, leading to distortion in the starch granule morphology. Thus, it can also explain why HAW starch is less regularly.

The swelling power of starch differed significantly in the three wheat varieties (Table 2). Xinong 836 had the highest swelling power (26.23 g g^−1^), followed by Zhengmai 7698 and Xinong 979. The results may be explained by the higher B-type starch content in Xinong 836. A positive correlation was also observed previously between the small SGs content and starch swelling power (30). In addition, we found that the water solubility of Xinong 836 (27.15%) was higher than those of Xinong 979 (23.73%) and Zhengmai 7698 (22.38%), mainly due to small SGs exhibiting higher hydrophilicity compared with large SGs.

### 3.5. Pasting properties and thermal properties of flour

The pasting properties of the wheat flour samples are shown in Table 3, Xinong 836 had the highest PV (1,622 cP) and BD (463 cP) values, and they differed significantly from those in its parents. The viscosity of flour is considered to be affected mainly by the properties of starch (21). Amylopectin is generally considered the main component responsible for the swelling of starch granules and increases in viscosity during heating, whereas amylose is often intertwined with amylopectin and it limits the swelling of SGs. However, an inhibitory effect of amylose on the pasting properties was not found in the present study. The high amylose content of Xinong 836 did not result in a decrease in the PV, possibly because the pasting properties are affected by various factors, such as the size or volume of SGs, protein contents, and non-starch polysaccharides in flour. In addition, Xinong 836 had the highest FV and SB values, and similar results were obtained in other studies where the volume of B-type SGs was positively correlated with the FV and SB values (19). However, Table 3 shows that there were no significant differences in T_o_, T_p_, and T_c_ among the three varieties, but Xinong 836 has the lowest ΔH (140.67 J g^−1^). According to Wang et al. (31), a lower ΔH is related to the presence of fewer starch crystallites. Thus, Xinong 836 with higher amylose content is accompanied by changes in crystallinity and also affects the thermal properties of flour.

**Table 3:** Pasting properties and thermal parameters of flour produced from Xinong 979, Zhengmai 7698, and Xinong 836.

Variety	Pasting properties					Thermal parameters			
	PV (cP)	BD (cP)	FV (cP)	SB (cP)	PT (°C)	T_o_ (°C)	T_p_ (°C)	T_c_ (°C)	ΔH (J/g)
Xinong 979	1245.00 ± 8.49c	446.00 ± 6.36a	1607.67 ± 10.61c	808.00 ± 8.49c	88.35 ± 0.57a	41.83 ± 0.49b	88.30 ± 1.39a	135.27 ± 0.49a	153.50 ± 6.36a
Zhengmai 7698	1408.67 ± 14.85b	391.00 ± 5.66b	1910.67 ± 14.85b	894.00 ± 6.36b	88.83 ± 0.04a	43.53 ± 0.87a	87.13 ± 2.23a	135.50 ± 1.61a	142.00 ± 5.57b
Xinong 836	1622.00 ± 8.48a	463.00 ± 5.66a	2085.33 ± 9.90a	926.00 ± 12.73a	88.70 ± 0.00a	43.77 ± 0.94a	87.17 ± 1.26a	136.00 ± 0.36a	140.67 ± 4.04b

^1^Values are expressed as mean ± standard deviation (*n* = 3). Results followed by a different letter in the same column are significantly different (*p* < 0.05).

^2^PV, peak viscosity; BD, breakdown viscosity; FV, final viscosity; SB, setback viscosity; PT, pasting temperature.

^3^To, onset temperature; Tp, peak of gelatinization temperature; Tc, conclusion temperature; ΔH, enthalpy of thermal transition.

### 3.6. Mixing properties of dough samples

The Mixolab2 results presented in Table 1 show that Xinong 836 had the longest development time (7.16 min) and stability time (9.55 min), followed by those for Xinong 979 and Zhengmai 7698. The mixing properties are responsible for the dough quality and they can be used as important indexes for the final product. According to a previous study, the dough made from waxy and HAW flour is less stable, thereby suggesting the presence of a weaker gluten network (32). Botticella et al. (33) also reported that the dough stability of HAW was significantly lower. However, we found that the mixing properties were not reduced for Xinong 836. Coincidentally, according to McCann et al. (14), high amylose content can significantly increase the ability of wheat flour dough to resist deformation and improve dough rheological properties. Interestingly, in McCann’s study, several wheat flours were formulated by the addition of HAW and/or commercial wheat starch as well as vital gluten to HAW flour. This maybe suggests that gluten cannot be ignored for the HAW with an excellent dough property.

### 3.7. Observations of dough microstructure

Fresh dough samples prepared from the three wheat varieties were quantitatively analyzed using CLSM and AngioTool software, as shown in Figures 2D–I and Supplementary Table S1. The dough samples produced from the three wheat varieties all contained dense and compact microscopic networks. Supplementary Table S1 shows the quantitative analysis results, which demonstrate that there were no significant differences in the protein area, protein junction density, and total protein length among the samples. However, Xinong 836 had the smallest lacunarity and it differed significantly from that in Xinong 979. The lacunarity represents the number of gaps and irregularities in a protein network, but it is also considered to reflect the size distribution of SGs in natural dough. According to a previous study, a higher lacunarity value is related to an irregular and less compact gluten network structure (9). The lacunarity was smaller in Xinong 836 compared with the other varieties, thereby indicating that it had a more compact network structure.

### 3.8. Synergistic effect of gluten and starch on dough properties

Through the above study, we found that the gluten structure, starch properties and dough properties of three wheat varieties showed great differences. As a mixture, wheat flour form dough by the complex interaction between gluten and starch. Therefore, we established the relationships among the above parameters. The correlation coefficients (r) obtained between the gluten structure, starch properties, and dough-related parameters are summarized in Table 4. The intermolecular β-sheets and disulfide bond contents were positively correlated with the dough development and stability time, whereas the α-helix and free sulfhydryl group contents were negatively correlated with dough behavior. These results are consistent with those reported previously (3, 15). The quality of the gluten protein network affects the strength and extensibility of dough. Xinong 836 had the highest β-sheets content and lowest α-helix structure content, and thus showed superior mixing properties. Similarly, positive correlations were found between the gluten structure parameters and BV. The BV value is an important indicator of the thermal stability of flour (3). Our results demonstrate that superior gluten structure is beneficial for enhancing the resistance of flour paste to shear force.

**Table 4:** Correlations between gluten structures, starch properties, and dough properties.

	Intermolecular β-sheets	β-sheets	α-helices	Sulfhydryl groups	Disulfide bonds	Amylose content	AC	BC	Relative crystallinity	Swelling power	Water solubility
DDT	0.930^**^	0.802	−0.946^**^	−0.909^*^	0.885^*^	0.783	−0.230	0.230	−0.167	0.066	0.800
DST	0.974^**^	0.785	−0.984^**^	−0.961^**^	0.951^**^	0.656	−0.078	0.077	0.000	−0.101	0.734
Lacunarity	−0.368	−0.746	0.407	0.237	−0.250	−0.890^*^	0.713^*^	−0.713^*^	0.780^*^	−0.794	−0.809
PV	0.067	0.528	−0.112	0.080	−0.068	0.769	−0.893^*^	0.893^*^	−0.984^**^	0.961^**^	0.689
BD	0.957^**^	0.801	−0.962^**^	−0.902^*^	0.890^*^	0.759	−0.150	0.149	−0.137	0.035	0.763
FV	−0.159	0.367	0.112	0.302	−0.290	0.619	−0.893^*^	0.893^*^	−0.998^**^	0.999^**^	0.532
SB	−0.245	0.308	0.196	0.391	−0.389	0.528	−0.853^*^	0.854^*^	−0.982^**^	0.992^**^	0.437
T_o_	−0.373	0.238	0.298	0.404	−0.433	0.474	−0.734^*^	0.734^*^	−0.779^*^	0.911^*^	0.445
△H	0.451	−0.225	−0.385	−0.491	0.392	−0.149	0.760^*^	−0.760^*^	0.748^*^	−0.893^*^	−0.450

^**^Correlation significant at *p* < 0.01. ^*^Correlation significant at *p* < 0.05. AC, A-type starch granule content; BC, B-type starch granule content; DDT, dough development time; DST, dough stability time; PV, peak viscosity; BD, breakdown viscosity; FV, final viscosity; SB, setback viscosity; T_o_, onset temperature; ΔH, enthalpy of thermal transition.

The A-type and B-type SGs contents were correlated with the dough lacunarity, thereby indicating that differences in the SGs contents could have affected the filling state of the dough. The presence of more small SGs contributes to a rather dense dough structure and affects the dough quality. The CLSM images also confirmed the compact structure (Figure 2I). In addition, the A-type SGs content was negatively correlated with the pasting properties of flour, whereas the B-type SGs content had positive correlations. These results indicate that the size of the SGs played an important role during flour gelatinization, mainly because small SGs can combine more readily with gluten protein to form a stable viscous phase in the gelatinization process (34). Moreover, the size distribution of SGs was positively correlated with thermal enthalpy, as also found in a previous study (1).

The relative crystallinity of starch was negatively correlated with PV (*r* = −0.984, *p* < 0.01), FV (*r* = −0.998, *p* < 0.01), SB (*r* = −0.982, *p* < 0.01), and T_o_ (*r* = −0.779, *p* < 0.05), but positively correlated with ΔH (*r* = −0.748, *p* < 0.05), thereby confirming that the relative crystallinity affected the pasting and thermal properties of flour. The swelling power and relative crystallinity had opposite effects on the flour pasting properties, mainly because the swelling power is related to interactions between amorphous and crystalline structures in starch, and negatively related to the relative crystallinity (35). The crystallinity is related to the hierarchical structure of double helices in SGs. SGs with a low-level helical structure are more likely to expose hydroxyl groups during heating, which can then combine with water through hydrogen bonds in the dough, thereby resulting in better dough behavior. In addition, SGs with lower crystallinity will form hydrogen bonds more readily with gluten glutamine (20), which helps to improve the interaction between starch and gluten to enhance the dough stability.

## 4. Conclusion

In this study, the grain development, gluten structural properties and starch properties of three varieties were investigated. We verified that gluten structure and starch properties are affected by the accumulation of gluten protein and starch, and the stability of gluten structure and characteristics of starch granules synergistically affected the pasting and thermal properties of flour paste. Xinong 836 with high amylose content has superior gluten structure, higher B-type starch content, lower starch crystallinity and lower enthalpy, which could be beneficial to improve the interaction between gluten and starch in the dough, and the presence of more small starch granules may contributed to dough with a rather dense structure, thus showing better dough property. In conclusion, our study demonstrates that superior gluten structure is a prerequisite for dough property. On this basis, the HAW starch which contains more small starch granules could lead to greater gluten-starch interactions and better dough property. Otherwise, more efforts should be initiated to create wheat that maintains a balance between the dough properties and the amylose content, rather than just increasing amylose content.

## Data availability statement

The raw data supporting the conclusions of this article will be made available by the authors, without undue reservation.

## Author contributions

LL, ZL, and XL: writing review and editing. XC: writing original draft. WY and BW: methodology. YX: project administration. XJL: writing review and editing. All authors contributed to the article and approved the submitted version.

## Funding

This study was supported by the Key Program of Shaanxi Agricultural Cooperative Innovation and Promotion Alliance (No. LMZD202104), the Special funds for Nanyang Wheat Experimental and Demonstration Station (No. 2021NY), and the Program of Introducing Talents of Innovative Discipline to Universities (Project 111) from the State Administration of Foreign Experts Affairs (No. #B18042) “Crop breeding for disease resistance and genetic improvement.”

## Conflict of interest

The authors declare that the research was conducted in the absence of any commercial or financial relationships that could be construed as a potential conflict of interest.

## Publisher’s note

All claims expressed in this article are solely those of the authors and do not necessarily represent those of their affiliated organizations, or those of the publisher, the editors and the reviewers. Any product that may be evaluated in this article, or claim that may be made by its manufacturer, is not guaranteed or endorsed by the publisher.

## Abbreviations

HAW: high amylose wheat
HMW-GSs: high-molecular-weight glutenin subunits
LMW-GSs: low-molecular-weight glutenin subunits
PBs: protein bodies
SGs: starch granules
DAA: days after anthesis
CLSM: confocal laser scanning microscopy
FTIR: Fourier transform infrared
XRD: X-ray diffraction
SH: free sulfhydryl
S-S: disulfide bond
