# Supplementary material for: Superior gluten structure and more small starch granules synergistically confer dough quality for high amylose wheat varieties
Source: Front Nutr. 2023 May 17;10:1195505. doi: 10.3389/fnut.2023.1195505 (PMC10230047; doi:10.3389/fnut.2023.1195505)
Supplement: Supplementary file 1 [file Data_Sheet_1.docx]

| **Table S1**  Parameters determined by AngioTool for the protein network in dough obtained from three wheat varieties. | | | | | | | | | |
| --- | --- | --- | --- | --- | --- | --- | --- | --- | --- |
| Variety | Protein area  (× 10^4^ μm^2^) | Protein percentage area (%) | Protein junctions (× 10^2^) | Junction density (× 10^–3^) | Total protein length (× 10^3^ μm) | End–points  (× 10^2^) | Lacunarity  (× 10^–2^) | Branching rate (× 10^–3^) | End–point rate (× 10^–3^) |
| Xinong 979 | 11.57 ± 0.48a | 44.37 ± 1.84a | 1.26 ± 0.10a | 4.84 ± 0.37a | 22.87 ± 0.86a | 3.91 ± 0.47a | 4.97 ± 0.37a | 1.09 ± 0.05a | 0.34 ± 0.05a |
| Zhengmai 7698 | 11.42 ± 0.28a | 43.76 ± 1.10a | 1.16 ± 0.04a | 4.43 ± 0.14a | 22.23 ± 0.25a | 3.13 ± 0.25b | 4.58 ± 0.19ab | 1.01 ± 0.02b | 0.28 ± 0.02b |
| Xinong 836 | 11.69 ± 0.19a | 44.79 ± 0.75a | 1.19 ± 0.05a | 4.56 ± 0.18a | 22.75 ± 0.36a | 3.05 ± 0.41b | 4.32 ± 0.43b | 1.02 ± 0.05b | 0.26 ± 0.04b |
| Values are expressed as mean ± standard deviation (n = 10). Results followed by a different letter in the same column are significantly different (*p* < 0.05). | | | | | | | | | |
