# Supplementary figures and images for: Superior gluten structure and more small starch granules synergistically confer dough quality for high amylose wheat varieties
Source: Front Nutr. 2023 May 17;10:1195505. doi: 10.3389/fnut.2023.1195505 (PMC10230047; doi:10.3389/fnut.2023.1195505)

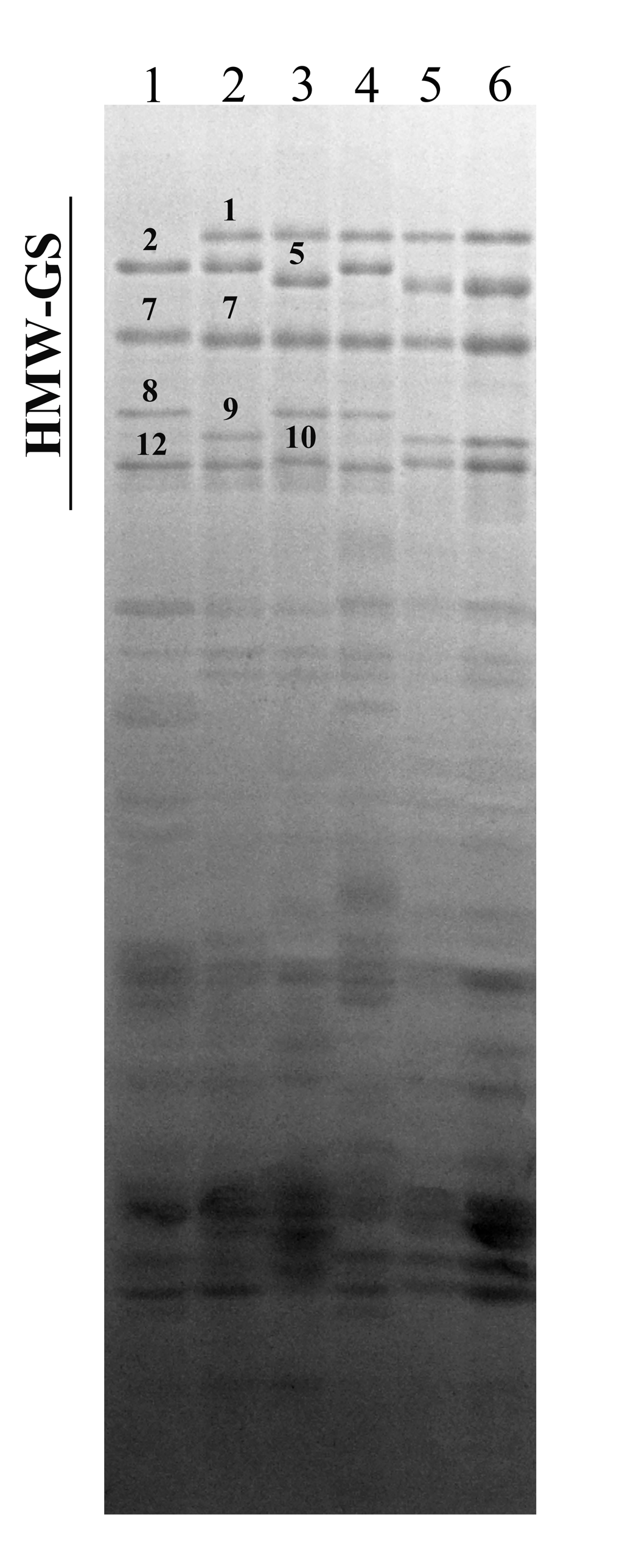

Supplement: Supplementary file 2 [file Image_1.jpg]

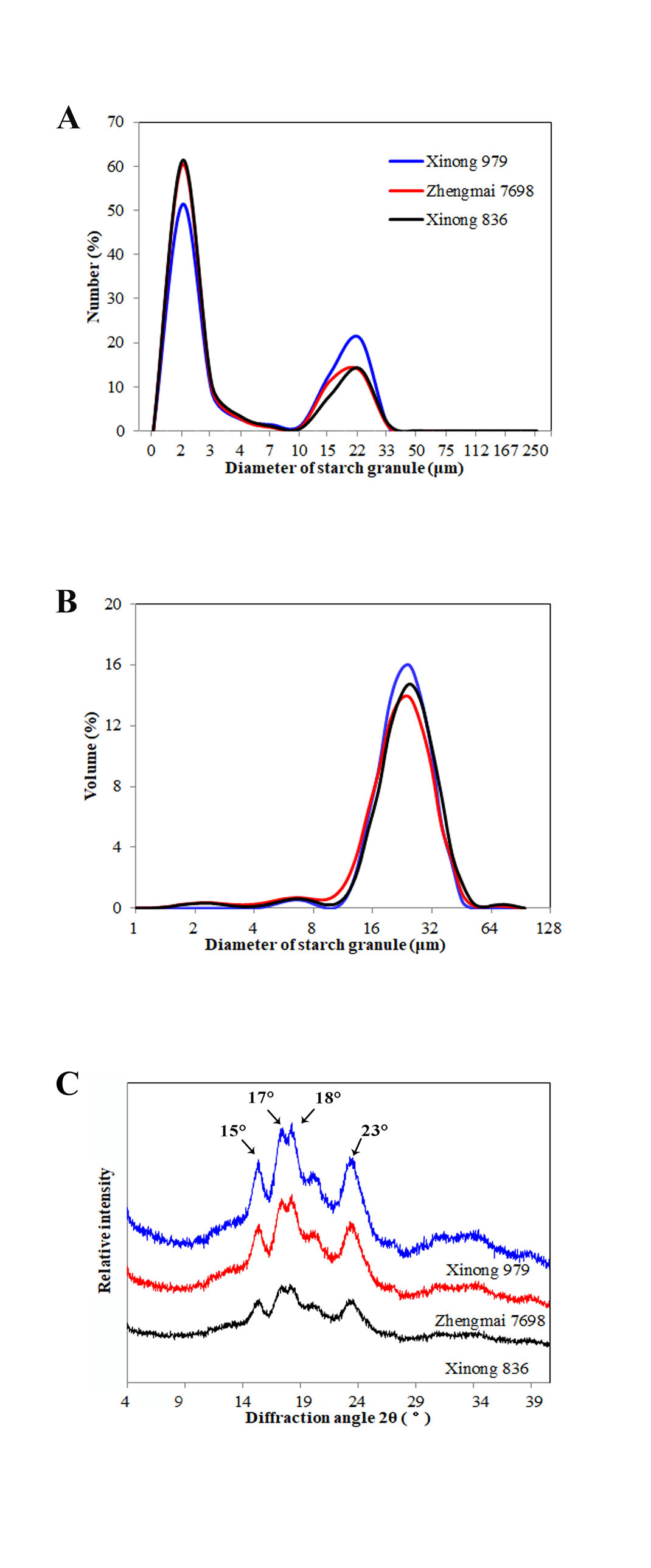

Supplement: Supplementary file 3 [file Image_2.jpg]

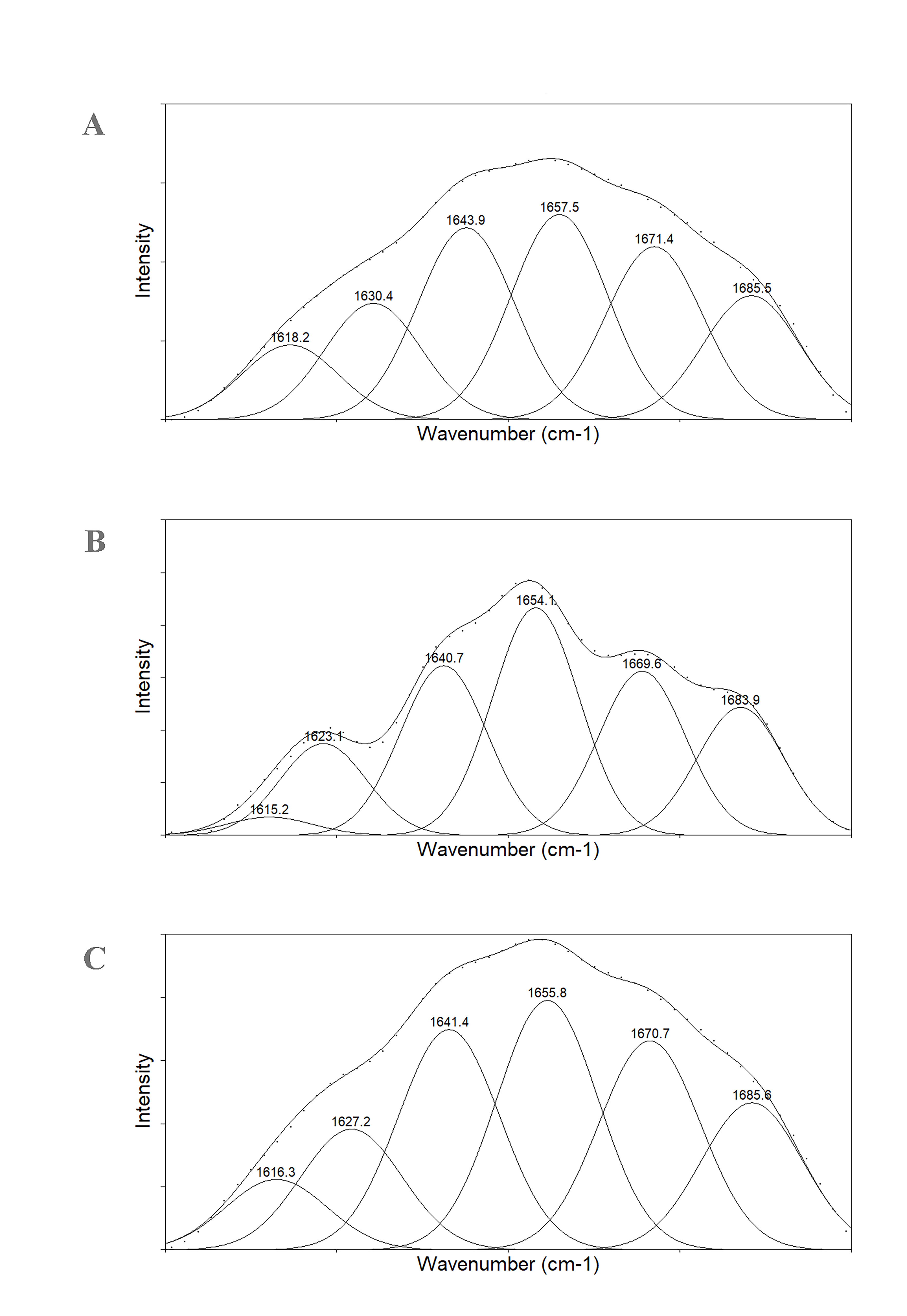

Supplement: Supplementary file 4 [file Image_3.jpg]

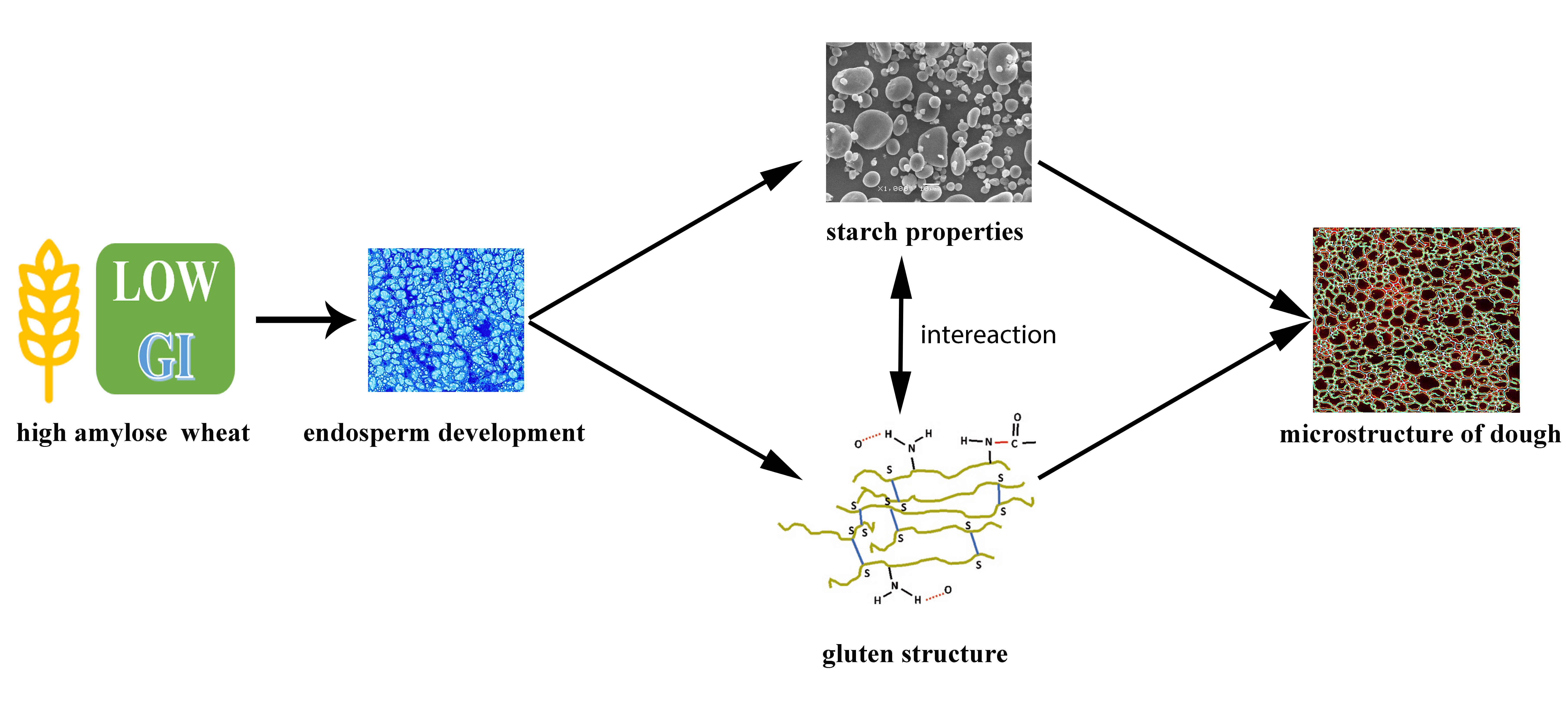

Supplement: Supplementary file 5 [file Image_4.TIF]
